# Supplementary figures and images for: Do mothers who delivered at health facilities return to health facilities for postnatal care follow-up? A multilevel analysis of the 2016 Ethiopian Demographic and Health Survey
Source: PLoS One. 2021 Apr 7;16(4):e0249793. doi: 10.1371/journal.pone.0249793 (PMC8026072; doi:10.1371/journal.pone.0249793)

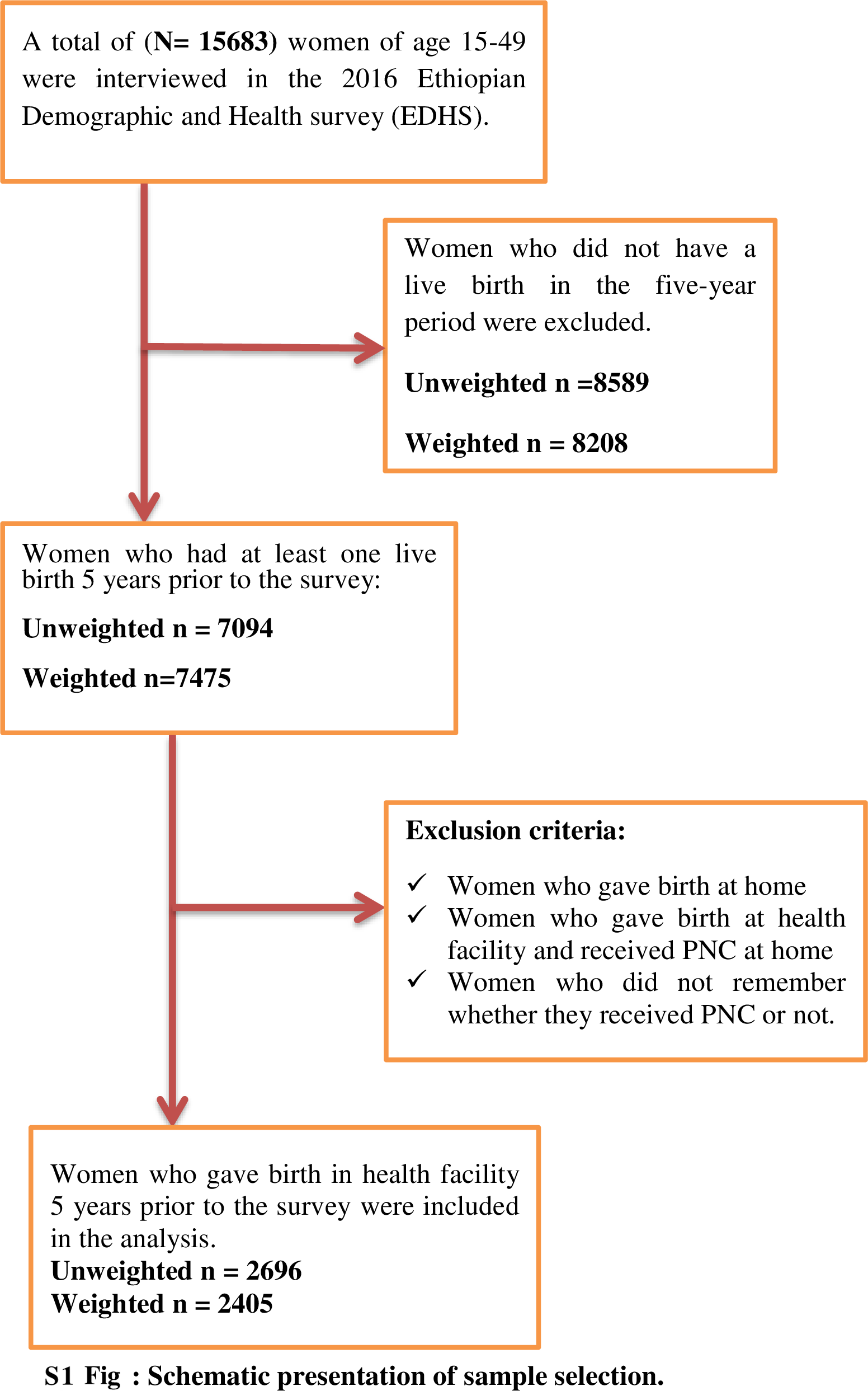

Supplement: S1 Fig — (TIF) [file pone.0249793.s001.tif]

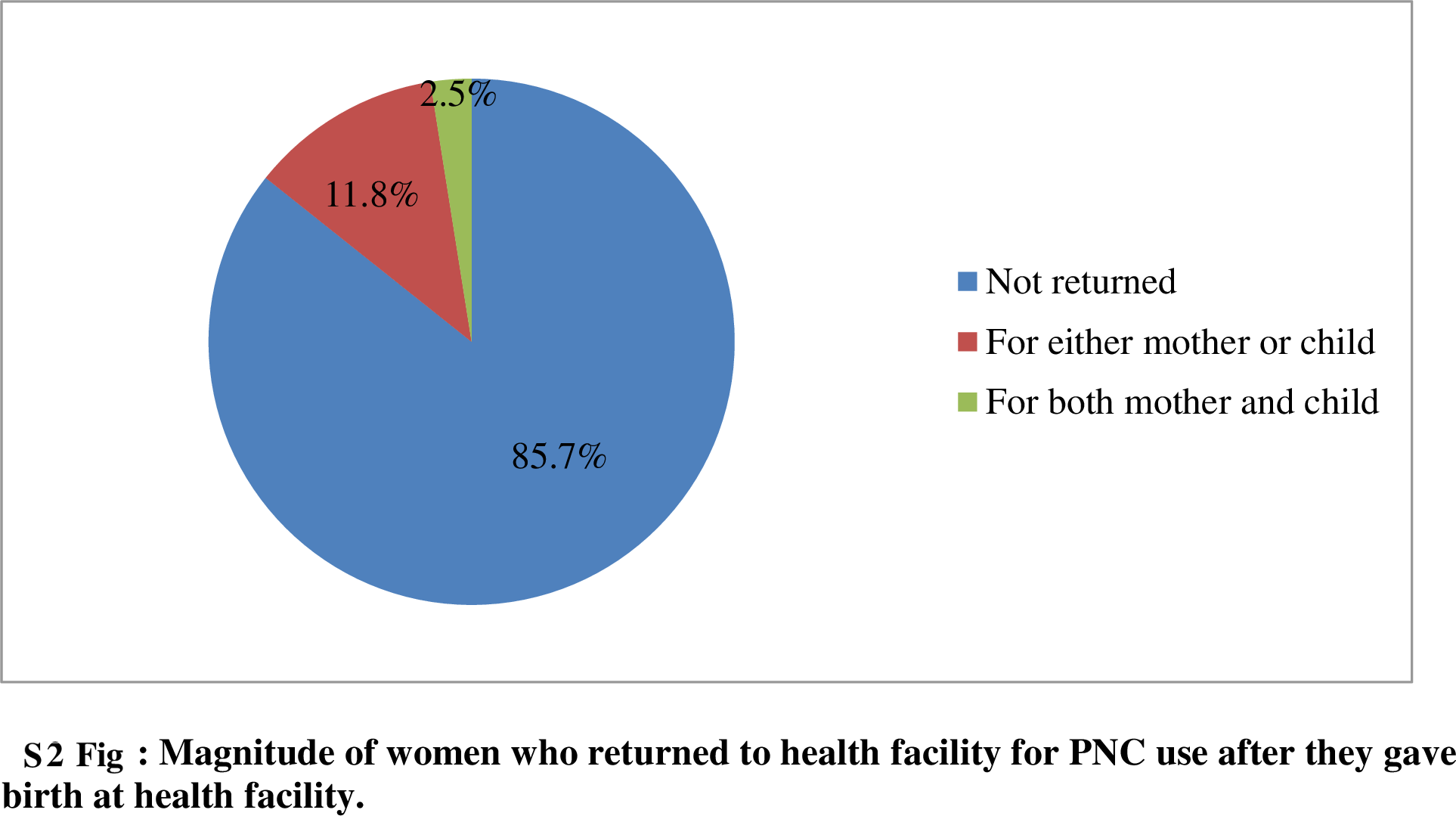

Supplement: S2 Fig — (TIF) [file pone.0249793.s002.tif]
