# Supplementary material for: Do mothers who delivered at health facilities return to health facilities for postnatal care follow-up? A multilevel analysis of the 2016 Ethiopian Demographic and Health Survey
Source: PLoS One. 2021 Apr 7;16(4):e0249793. doi: 10.1371/journal.pone.0249793 (PMC8026072; doi:10.1371/journal.pone.0249793)
Supplement: Attachment — Submitted filename: Response to reviewers.docx [file pone.0249793.s003.docx]

**Response to reviewers**

Dear editor and reviewers thank you very much for your comments and suggestions. Your concerns are very important and critical to improve our manuscript accordingly. We tried to address the points in our revised manuscript. Here are our point by point responses!

| **Comments** | **Authors response** |
| --- | --- |
| **Editor’s Comments** |  |
| At least three published studies (Sisay et al, 2018; Ayele et al 2019; Fikadu et al, 2019) have so far identified predictors of PNC utilization in Ethiopia based on the same dataset (Ethiopian DHS 2016)bused in this study. What was the justification for reanalyzing the dataset again? | Thank you for the comment and for raising the issue. Dear editor, though we make it clear in the current manuscript, the reason that we analyze the data is that our study population in our analysis and the study population in the mentioned studies are different. For example, the study population in Sisay et’al, 2018 was any women in childbearing age that gave birth in the last 5 years preceding the survey in the selected EAs irrespective of place of delivery. The study population in Ayele et’al, 2019 was also women in child bearing age that gave birth in the last 5 years preceding the survey and delivered at home. The study population in Fikadu et’al, 2019 was mothers who delivered at health facility and only those who had fourth antenatal care visit. Furthermore, there is also a recently published study (Tiruneh, 2020) on postnatal care utilization with a study population of women who give birth in both health facility and home.  Thus, as our study is only included women who gave birth in health facilities it is different from all the above mentioned four articles. |
| The operational definition used for postnatal care (any health check-up in the first 42 days) is deficient because it does not take the timing and number of PNC visits into consideration. At least basic description on the timing and frequency of PNC visits must be given in the manuscript. | If I understand your concern, you expect us to explain the timing and frequency of PNC visit. Thank you! However in this study the main objective that we want to explain is not the frequency and timing of PNC. We need to show how much mothers (those who delivered in health facilities) returned to any health facility (could be governmental, private, to the same facility where they gave birth or to other health facility) at least once before their 42 days of postpartum period. That’s why we did not include the frequency and timing of the visit. |
| The authors provided inadequate information how they analyzed the data. As the DHS employed cluster sampling design, data must be analyzed using analytical techniques (e.g. mixed effects model, GEE or survey design) that accommodate the clustering nature of the study. Furthermore, sampling weights provided in the data and post stratification weight for balancing the contribution of the regions must be used. | Thank you very!  The data analyzing method is changed according to your comment. Thus, we used mixed effect model to analyze the data so as to accommodate the clustering of the data. Furthermore, we also used sampling weigh to balance the regional variation. Thank you once again! |
| The reason for including some of the variables included in the multivariable is not clear. The variables “Children born in last five years” and “Children born in last one year” are likely to have redundant information in the model and hence one of them has to be dropped. Further, while analyzing these variables, the authors should rather be focused on number of births that occur before the index pregnancy analyzed for the PNC. Births that occur after the index birth should be excluded and this can easily be done in DHS by taking date of birth into consideration. | Acceptable comment. The analysis already changed and this comment is taken it account. Thank you very much for your concern. |
| The variable “Currently working” is also confusing. Does it refer to the occupation of the mother during the index pregnancy? How can the current occupation affect PNC received some years back? | Thank you! However, DHS data collects the socio demographic characteristics (like age, educational level, working status and wealth status) of the participants during the data collection. They can’t be collected in relation to specific event. That’s why we took the work status. However, to prevent confusion among readers we prefer the “Occupation” to “Currently working”. |
| Two variables (ownership of mobile phone and having bank account) are already used for developing the wealth index. So, what is the purpose of considering them as distinct variables again? | Thank you! Acceptable and these variables are omitted in this manuscript. |
| It is not clear how 50% of the data fall into the richest wealth quintile (Table 1). As I understand, wealth index in DHS data divides the study subjects into 5 equal Quintiles each nearly having 20% of relative frequency. I recommend the authors to redo the PCA again. | Method of analysis is changed and your concern is already solved. Thank you very much! |
| Fitting 18 variables in one model would be too much and might result in an unstable model. I recommend the authors to remove less relevant variables or to use more stringent p-value for screening candidate variables for the multivariable model. | In the revised manuscript 15 variables are fitted in the mixed effect multilevel logistic regression model. Thank you for your valuable concern! |
| **Additional Editor’s Comments (Section-by-section comments):** |  |
| Abstract   - The operational definition for “utilization of PNC” should be provided - The conclusion sub-section should be condensed and should not repeat what has been reported in the results. | Thank you!  The introduction and the conclusion of the manuscript are revised according to your suggestions. |
| Background   - Please don’t direct copy from other literature. Many sentences and paragraphs have not been adequately paraphrased. | Paraphrasing is conducted as much as possible. Thank you! |
| Methods   - Please describe the basic sampling technique employed in the DHS survey. | Acceptable comment and basic sampling technique of the DHS survey is included in the revised manuscript. Thank you! |
| - Line 92-93: “Only the most recent child of the women was included in the analysis”. Please clearly indicate that this is about the most recent birth that happened in the preceding 5 years of the survey. | Thank you! The comment is acceptable and included. |
| - Figure 1: I think the decision to exclude women who had home PNC is wrong. The data of these subjects should be retained in the analysis but they should be considered as they have no PNC. | Thank you for the comment!  However, women who received PNC at home were excluded due to the reason that the interest of this manuscript is mainly to show the health seeking of mothers for PNC after they gave birth in health facilities which could be decreased if they received it in home. In other words why they need to return to health facility for PNC reason if they received it in their home? They don’t! That’s why we exclude them from the analysis. We revised the figure to make it more clear in the revised manuscript. |
| Results   - Socio-demographic and socio-economic characteristics: Looks very superficial. Please expand - Please interpret OR>1 on multiplicative, rather than additive scale. - Table 3: “pace of delivery” >> “place of delivery” | Thank you very much! In the revised manuscript all of these comments are considered! Please see the revised version of the manuscript. |
| Discussion   - Please integrate the Strength and Limitation section with the Discussion section. - Paragraph 2: is just a repetition of the findings of the study, I don’t see any discussion - Paragraph 3: Comparison should only be made with studies that estimated PNC coverage among women who had health facility delivery. | - Thank you for the comment. As you said in the strength and limitation part is integrated with the discussion part. - The second paragraph is the repetition of the result. Yes, this part (the second paragraph) is the summary of the finding which is important to grasp the attention of the readers what the key findings of the study and their discussion points are followed subsequently. - This is acceptable comment |
| **Reviewers comments** |  |
| Reviewer #1: Thank you for this well written manuscript. Just a few minor comments and questions:  Outcome variable  • Line 108: Please clarify whether the PNC being discussed is for the mother, the child or both | First I thank you very much for your precious comments!   - The outcome variable which is “Return to health facility for PNC” in the revised manuscript is for both the mother and the baby. It ‘yes’ if the mother returned to use PNC either for her baby or herself or both. This is clearer in the revised version of the manuscript. |
| • It is not quite clear to me what is meant by “follow up check”. Is the paper focusing on immediate postnatal care or any postnatal care? Follow up would essentially mean that the mother got a first check up, and then they had a follow up check after a few days/weeks? Is this the case, or are the investigators only considering the first check? This needs to be  clarified both when discussing the derivation of the outcome variable but might also need to be reflected in the topic | Thank you!  The paper’s focus is on PNC seeking among women who delivered in health facility; we do not focus on whether they received PNC before discharge or not. We only focus on ‘do mothers returned for PNC use to health facilities after they gave birth in health facilities at least once in their postpartum period?’ which can show the health seeking behavior of mothers for PNC. To make it more clear your comment is very important and we amend the title of the manuscript as well considering your suggestion. Thank you once again! |
| • At some point the authors have mentioned that women who were checked within a month were considered to have accessed PNC. However, the WHO guidelines stipulate that PNC should be accessed in the first 42 days after birth. Can the authors please clarify this, or correct this in their generation of the outcome variable? | If I understand your comment well, as you said the PNC should be accessed in the first 42 days after birth with a recommended frequency of four. Thus, in our manuscript we also took this period in to account regardless of the frequency; whether mothers were returned for PNC use after they gave birth at health facilities. Furthermore, the PNC for babies was considered up to two months after birth for explained reason. |
| Statistical analysis  • Demographic and Health Surveys are usually sampled at two levels. The authors have not mentioned that they adjusted their analysis for sample weights. How did the authors account for the multilevel structure of the data? | Thank you very much!  All your comments are acceptable and the revised version of the manuscript includes considerations to the mentioned (sampling weight and multilevel structure) issues. |
| • Line 91: DHS does not have a specific data set for pregnancy and postnatal care. Can the authors clarify in the write up whether they used the birth recode or the women’s recode? This will be useful for reproducibility of the results by the readers. The DHS does not have a unique file for pregnancy and postnatal care | Thank you your comment is acceptable and included in the revised manuscript. The “Individual Record (IR) dataset was used! |
| • Line 115 and 116: I would expect the following variables to be highly collinear: (number of ever born children, children born in last five years, children born in last one 116 year). Is there a particular reason why you chose to include all three?  What was the VIF for these variables? | Thank you!  In the revised version your concerns are addressed! Unfortunately these variables are not included in the new version due to the reason that these variables were not fulfilling the assumption for inclusion. |
| • What bivariate method was used? This should be specified in the write up. I have not seen results of any bivariate analysis presented. What the authors have presented is a univariate logistic regression analysis. | Thank you!  Bivariable multilevel logistic regression was used and clearly stated in the revised manuscript. |
| • In the methods section there is a mention of the VIF, but I don’t see the results from the VIF being talked about anywhere. Were any of the variables highly correlated or not? A sentence on this might be useful when starting to discuss results from the logistic regression | Thank you!  VIF was done and no variables were above 5 and this is clearly stated in the revised document. |
| Table 1  Ownership of pace of delivery should have place of delivery instead of pace | Thank you!  Unfortunately this variable is omitted in the revised version as we only put variables which fulfill the assumption for inclusion. |
| Table 2  • Other(single, widowed, divorce): divorce should be divorced to be consistent with the other words | Corrected! |
| Table 3  • Part 3 of the caption of Table 3 should read as a table that is continuing from the previous pages. Just as it was done on  the second part of the table | Corrected! |
| • P-values are usually categorized as follows: *** p-value <0.001, ** p-value < 0.01, * p-value < 0.005. The authors might  wish to adopt this on the same p-value note, | Thank you for your suggestions! However, as the result of p-value in the final model rages from 0.002 to 0.04 for significant variables, we adapt P-value ≤ 0.005** and 0.05* respectively. |
| • Why is it that the statistically significant results in the adjusted logistic regression model have been highlighted but those in the unadjusted model have not? | Corrected! |
| • I find Table 3 to be a little crowded. Firstly, the table caption says that the table presents results from the logistic  regression, and yet the table includes results from cross-tabulations  • Normally, the cross tabulations would be included in Table 1. i.e. one could have 3 columns in the table: had PNC (n & %), did not have PNC (n & %), total (which is what is currently the column in Table 1 (n & %)) | Thank you very much! It is acceptable comment and corrected accordingly. Please see the revised version of the document. |
| Reviewer #2: 1. The manuscript is technically sound because it covers all the aspects of a good manuscript and content stuck to the subject matter. The data supports the conclusion methodology; results (proportions and AOR) appropriately handled and presented to draw the conclusion.  2. Statistical analysis well handled - explanations of process and methods appropriately selected and handled.  3. The Author clearly indicated that the data will be made available without restrictions.  4. Lines 69 and 70, the sentence is not clear, "not advised to return to more complications among mothers". Line 81 consideration of removing the word despite and make the point more clear. Line 151 to rephrase the sentence and add - women who were not checked before discharge. | Thank you very much!   - The phrase "not advised to return to more complications among mothers" in line 69-70 is revised and we tried to make it more understandable. - The “despite” in line 81 is amended and we tried to make it clear. - Accepted and your concern is considered. |

Dear editor and reviewers, once again we thank you very much for all your valuable comments!

Kindly regards,

Brhane Ayele (on behalf of all authors)
