# Supplementary material for: Do mothers who delivered at health facilities return to health facilities for postnatal care follow-up? A multilevel analysis of the 2016 Ethiopian Demographic and Health Survey
Source: PLoS One. 2021 Apr 7;16(4):e0249793. doi: 10.1371/journal.pone.0249793 (PMC8026072; doi:10.1371/journal.pone.0249793)
Supplement: Attachment — Submitted filename: Response to reviewers.docx [file pone.0249793.s004.docx]

| **Comments** | **Authors ‘ response** |
| --- | --- |
| **Editor’s comments** |  |
| Please make sure that the manuscript is thoroughly edited for typographical and grammatical errors. | Thank you very much for the concern. As much as possible, we thoroughly edit for typographical and grammatical errors. |
| **Reviewer’s comments** |  |
| **Abstract**   - I am a Statistician, but have never actually heard the term “bi-variable multilevel logistic regression” before. What do the authors mean by this? Did you, perhaps, mean to say a univariate/unadjusted multilevel logistic regression? - Delete were in the sentence “In this analysis, from the total 2405 participants, 14.3% ((95%CI: 12.1-16.8), (n=344)) of them were returned to health facilities for PNC use after they gave birth at a health facility” | - Yes, we mean to “unadjusted multilevel logistic regression”. Dear reviewer it is common to say “bi-variable multilevel logistic regression” for unadjusted multilevel logistic regression considering the two variables (one dependent and one independent). However, to make it more clear we revised the manuscript according to your comments. Thank you very much! - Thank you very much! |
| **Introduction**  • Line from 54 to 56. Remained should probably be remains.  • Authors might wish to have someone check the grammar throughout the paper | Accepted! Thank you! |
| **Methods**  • Great job explaining how the outcome variable was derived and how the independent variables were defined  • I would also like to commend the authors for explaining their choice of methods in detail  • Authors should avoid using the term bivariable/bivariate multilevel logistic regression. The appropriate term is  univariate/unadjusted multilevel logistic regression. | Thank you for your kind recognition and comments; your comments and concerns are considered. |

**Response to reviewers**

Dear editor and reviewer thank you very much for your comments and suggestions on the revised version of our manuscript. As usual, your comments and concerns are very important and critical to improve the manuscript accordingly. We tried to address the points in this re-revised manuscript. Here are the point by point responses!

| **Results**  Socio-demographic and socio-economic characteristics  • Sentence 213: “were returned” should be returned. Can this be implemented throughout the manuscript, please  • Sentence 220: capitalize t on Table-1. Table should always have a capital T in the text | Accepted! Thank you! |
| --- | --- |
| Associated factors with return to health facility for PNC (fixed effects)  • Odds ratios are supposed to be reported together with their reference categories. For instance, sentence 241:  “Employed women were 51% (AOR=1.51, 95%CI: 1.04-2.19) more likely to return to health 242 facility for PNC during their postnatal period after they gave birth at health facilities” – more likely than who? The reference category should be mentioned for all the sentences explaining the odds ratios. This has been done for sentence “Women who delivered by caesarean section were also in higher odds (AOR= 2.53, 95%CI: 1.40-4.58) of returning to health facilities for PNC than their counterparts during their postnatal period.”, but not for the other sentences. | Thank you very much! Your concern is addressed in the re-revised version of the manuscript. |

Dear editor and reviewer, once again we thank you very much for all your valuable comments!

Kindly regards,

Brhane Ayele (on behalf of all authors)
